# Distressed (Type D) personality is predicted by avoidance: evidence from a computer-based avatar task

**DOI:** 10.7717/peerj.14302

**Published:** 2022-10-27

**Authors:** M. Todd Allen, Michelle M. Shields, Catherine E. Myers

**Affiliations:** 1 School of Psychological Sciences, University of Northern Colorado, Greeley, CO, USA; 2 Department of Pharmacology, Physiology & Neuroscience, Rutgers University—New Jersey Medical School, Newark, NJ, USA; 3 Department of Veterans Affairs, VA Medical Center, East Orange, NJ, USA

**Keywords:** Personality, Assessment, Social inhibition, Negative affect, Distressed personality, Type D personality, Avoidance

## Abstract

**Background:**

One personality type associated with poor health outcomes is distressed (Type D) personality which involves high levels of both social inhibition (SI) and negative affectivity (NA). Type D is also linked to psychopathologies such as post-traumatic stress disorder (PTSD), anxiety disorders, and depression. One mechanism through which personality temperament may result in these psychopathologies is avoidance. Recently, a computer-based measure designed to assess avoidant behaviors, in which the participant guides the behavior of an avatar interacting with strangers in social situations, has been found to be related to various forms of avoidance. In the current study, we extended this work with the avatar avoidance task to determine its relationship to distressed (Type D) personality. We hypothesized that Type D personality, along with SI, but not NA, would be positively related to avatar avoidance scores. We also hypothesized that avatar avoidance scores would be higher in Type D individuals than non-Type D individuals.

**Methods:**

A total of 302 undergraduates completed the Distressed Type D Personality Scale (DS-14), and a computer-based avatar avoidance task.

**Results:**

Type D and SI, and NA to a lesser degree, were positively correlated with avoidance scores on the avatar task. Furthermore, regression analyses revealed that Type D and SI scores were best predicted by a model including avoidance scores and education level while NA scores were best predicted by a model including avoidance scores. Standard cut-off scores on the DS-14 scale resulted in four groups (*i.e*., low SI and NA, high SI, high NA, and Type D) which significantly differed in avoidance scores. Specifically, Type D individuals had higher avoidance scores than the other three groups. Taken together these findings support a role for avoidance in Type D personality. The computer-based avatar avoidance task may be particularly relevant as an ecologically valid measure to identify avoidance in a virtual setting for use with individuals expressing Type D personality who may be unwilling or unable to accurately self-report or describe their own avoidant tendencies.

## Introduction

One personality temperament that has received attention in physical and psychological health is distressed (Type D) personality (Denollet, Sys & Brutsaert, 1995; Denollet et al., 1996). Type D personality is characterized by high levels of both negative affectivity (NA) and social inhibition (SI). Individuals classified as Type D exhibit trait-like tendencies to experience negative emotions but suppress or inhibit the expression of these emotions in social settings for fear of the reactions of others. Type D is assessed with the Distressed Type D Personality Scale (DS 14; Denollet, 2005) which consists of separate NA and SI subscales. Separately, NA and SI are considered continuous variables. Lodder et al. (2021) reviewed how Type D can be operationalized as either a dichotomous or continuous variable. Type D can be calculated either as the sum of the NA and SI scores (*i.e*., additive method) or as the product of NA and SI scores (*i.e*., synergistic method). Type D personality is generally considered dichotomous in that an individual either meets or does not meet the criteria of high levels of both NA and SI in order to be categorized as Type D (Kupper et al., 2013a). This dichotomous method can be extended by categorizing individuals into four groups consisting of those with low scores for both SI and NA, those with high scores for only SI, those with high scores for only NA, and those with high scores for both SI and NA (*i.e*., Type D). However, Kupper & Denollet (2016) indicated that Type D can also be analyzed as a continuous variable. Lodder et al. (2021) concluded that analysis of Type D as a continuous variable may reduce false positives resulting from the dichotomous method in which Type D status is based only high scores on SI or NA alone rather than their combined additive or synergistic effects.

Type D was developed as a general vulnerability factor for poor prognosis in patients with ischemic heart disease. The high levels of both NA and SI result in patients who are unwilling to seek out social support (Martens et al., 2007). Type D personality is associated with increased anxiety (Pedersen et al., 2004), depression in cardiac patients (Pedersen et al., 2004), undergraduates (Gupta & Basak, 2013), and military populations (Servatius et al., 2017) and post-traumatic stress disorder (PTSD; Mommersteeg et al., 2011; Oginiska-Bulik & Langer, 2007; Pedersen & Denollet, 2004; Rademaker et al., 2011; Servatius et al., 2017).

One aspect of Type D personality that may underlie its relationship to anxiety disorders, and PTSD is avoidance coping. While avoidance is a normally adaptive response in the face of stress or trauma, it can become maladaptive if it persists after the threat has ended or becomes so pervasive that it disrupts normal social interactions and life functioning. In general, avoidance contributes to the chronicity of PTSD or anxiety disorders as it prevents the individual from learning that certain situations or stimuli are not or no longer dangerous. There is evidence linking Type D personality with repressive coping (Denollet, 2005) and avoidance coping (Polman, Borkoles & Nicholls, 2010; Dunkley et al., 2012). Specifically, Polman, Borkoles & Nicholls (2010) reported that Type D individuals exhibit more passive and maladaptive avoidance coping strategies such as resignation and withdrawal while being less likely to utilize more adaptive approach coping mechanisms. These avoidant coping strategies also mediated the relationships between Type D and perceived stress and burnout (Polman, Borkoles & Nicholls, 2010). More specifically, avoidance coping mediated the relationship between Type D and physical symptoms and emotionally-based coping mediated the relationship between Type D and perceived stress (Williams & Wingate, 2012).

SI is also related to avoidance symptoms of PTSD (Rademaker et al., 2011). SI is considered a multidimensional construct which includes behavioral inhibition, interpersonal sensitivity, and social withdrawal (Denollet & Duijndam, 2019). Behavioral inhibition (BI) is defined by a temperamental tendency to withdraw from or avoid novel social and non-social situations (Kagan, Reznick & Snidman, 1987; Morgan, 2006). A series of recent studies have supported a learning diathesis model whereby BI is put forth as a risk factor for PTSD (see Allen et al., 2019). This work has included a limited examination of Type D and BI. Specifically, BI as measured by the Adult Measure of Behavioural Inhibition (AMBI; Gladstone & Parker, 2005) was found to have a strong positive relationship with Type D as well as moderate positive relationships to SI and NA in a sample of undergraduates (Allen et al., 2018) and active-duty military expressing PTSD symptoms (Servatius et al., 2017).

Given these relationships between BI and Type D personality, the current study sought to investigate the relationships of Type D to a newly developed a computer-based task which was designed as an alternative to paper and pencil measures of BI such as the AMBI. Myers et al. (2016a) developed a computer-based task in which participants select an avatar that they then guide through social interactions with strangers while indicating how they would respond to these interactions in real life. As a series of studies have demonstrated, performance on this avatar avoidance task is positively related to BI (Myers et al., 2016a), avoidant symptoms of PTSD (Myers et al., 2016b), harm avoidance (Allen, Jameson & Myers, 2017), behavioral avoidance, but not cognitive avoidance (Allen, 2018), and avoidant coping styles (Allen & Myers, 2019). Overall, avoidance on this avatar task has been found to have highly significant positive relationships with a variety of avoidant personality factors suggesting that assessments of behavior in virtual environments can assess avoidant aspects of personality. Thus, the current study utilizes the avatar avoidance task to explore the role of avoidance in Type D personality.

As previously described, Type D can be operationalized as either a continuous or dichotomous variable. In the current study, we examined Type in both manners. First, we explored the relationships of Type D as a continuous variable with avoidance as measured by the avatar task. Based on the strong relationship between Type D and BI as well as a strong relationship between performance on the avatar task and BI and other avoidant personality temperaments, we hypothesized that performance on the avatar task would have a strong positive relationship to Type D, as well as SI. However, we hypothesized that avatar avoidance scores should not be related to NA based on the fact that none of the scenarios in the avatar task target depressive behaviors. Second, we examined Type D as a dichotomous variable by grouping individuals into four groups consisting of Type D individuals with high scores for both SI and NA, those with only high scores for SI, those with only high scores for NA, and those with low scores for both SI and NA. We hypothesized that avatar avoidance scores would be higher in Type D individuals than non-Type D individuals.

Another variable of interest in the current study is gender. Reports of gender effects for Type D, SI, and NA have been inconsistent. Several studies examining Type D with various health issues across several cultures have not demonstrated any gender-specific effect (*e.g*., Denollet, 2005; Williams et al., 2008; Barnett et al., 2009) However, some studies have reported higher rates for Type D in females as compared to males (Grande et al., 2004; Kupper et al., 2007; Denollet, Vrints & Conraads, 2008; Pedersen et al., 2009; Mommersteeg et al., 2011; Kupper et al., 2013b; Staniute et al., 2015; Cho & Jeon, 2018). Reports of gender effects for SI and NA have been mixed. For example, some studies found that females have higher SI than males (Gebska et al., 2021; Duijndam et al., 2020) while Kupper et al. (2013b) reported that males have higher SI levels than females. Some studies have also found that females have higher NA than males (Grande et al., 2004; Kupper et al., 2013b; Gebska et al., 2021). However, other studies have reported no gender differences for SI or NA (Vukovic et al., 2014). Results have been more consistent for performance on the avatar task in that no studies have reported a gender effect (Myers et al., 2016a, 2016b; Allen, Jameson & Myers, 2017; Allen, 2018; Allen & Myers, 2019). Therefore, it would be of interest to examine possible gender differences in Type D, SI, NA and their relationships to avoidance scores on the avatar task.

## Materials and Methods

### Participants

Three hundred and two participants including 202 females and 100 males voluntarily participated in this study in exchange for partial credit for a research requirement in an introductory undergraduate psychology course. Participants had a mean age of 19.5 years (*SD* = 4.0 range = 18 – 56) and a mean education level of 12.9 years of schooling (*SD* = 1.2). Race/ethnicity of the participants was self-reported as Caucasian (*n* = 194), Hispanic (*n* = 62), African-American (*n* = 13), East Asian (*n* = 5), South Asian, (*n* = 1), multi-racial (*n* =21), and other (*n* = 6).

### Instruments

#### Distressed Type D personality scale

Participants completed the Distressed (Type D) Personality Scale (DS-14; Denollet, 2005) which includes seven items assessing social inhibition (SI) and seven items assessing negative affectivity (NA). Participants rated each item as to whether it was true or false for him/her on a range of five options including false (0 points), rather false (1 points), neutral (2 points), rather true (3 points) and true (4 points). A sample item for assessing SI is “I would rather keep other people at a distance.” while a sample item for NA is “I am often in a bad mood.” Scoring of the DS-14 inventories were done based on published methods. Based on the methodology of Kupper & Denollet (2014), Type D was calculated as a synergistic interaction term (NA * SI). We also used the standard cut-off scores in which individuals with scores of 10 or higher on both the NA and SI subscales were categorized as Type D (Denollet, 2005). Individuals with scores of 10 or higher on the SI or the NA subscales were categorized as high SI or high NA, respectively. Individuals with scores less than 10 for both the SI and NA scales were categorized as low both SI and NA. The authors have received permission to use this instrument from the copyright holders.

The DS-14 has strong psychometric properties including an overall internal consistency of Cronbach’s alpha of 0.88 for the NA subscale and 0.86 for the SI subscale. A sample of 121 patients tested across a 3-month interval revealed a test retest reliability of 0.72 and 0.82 for the NA and SI subscales, respectively (Denollet, 2005). The current study had a Cronbach’s alpha of 0.87 for the NA subscale and 0.84 for the SI subscale.

#### Computer-based avatar task

**Figure 1:**
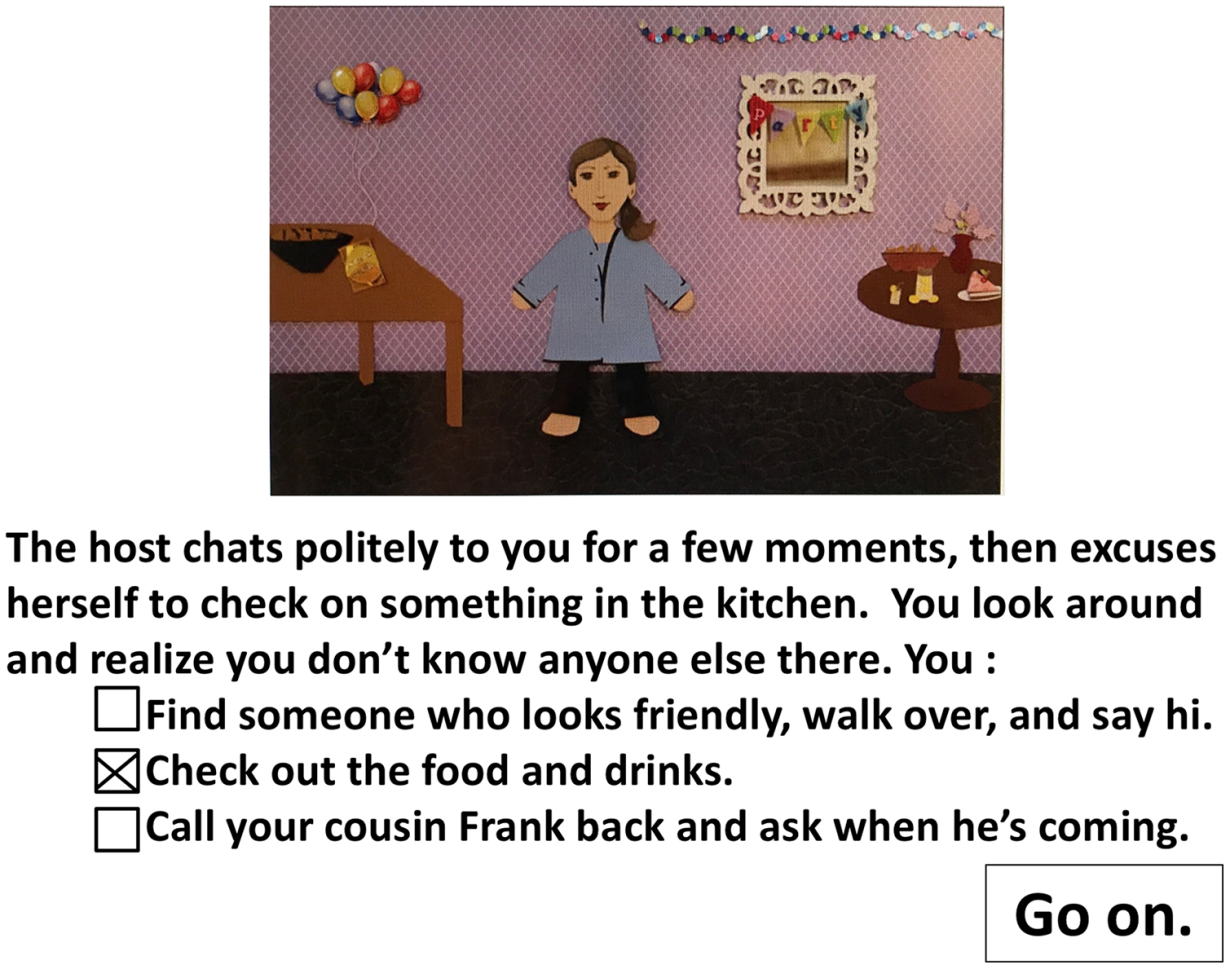
Screenshot of the avatar avoidance task. An example of a choice point from the computer-based avatar task. In the party scenario, the avatar was invited to a party where she did not know anyone there except her cousin who had yet to arrive. In the scenario, the avatar has a choice between a non-inhibited response, an intermediate response, and an inhibited response. In this case, the intermediate response was chosen.

An open-access version of the avatar task software is available online at Open Science Framework: www.osf.io/zf3jv. In brief, participants first selected an avatar from eight avatars which included a variety of hair and skin colors. The participant then guided the avatar through two scenarios, attending a party and volunteering with a charity building project. Both of these scenarios involved interactions with strangers. The script included twenty decision points where a still image appeared on the screen in which the avatar experienced a social interaction along with a short text description and three response options (*e.g*. Fig. 1). The response options always included a relatively avoidant response (worth 2 points), a relatively non-avoidant response (worth 0 points), and an intermediate/neutral response (worth 1 point). The response items and point values were based on the structure of the Adult Measure of Behavioural Inhibition (AMBI; Gladstone & Parker, 2005). All participants experienced the same scenarios regardless of their responses. Total scores could range from 0 to 40, with higher scores indicating higher avoidance. The point values and the total scores were not visible to the participant. The avatar task has good inter-item reliability as indicated by Cronbach’s alphas of 0.78 to 0.98 (Myers et al., 2016a, 2016b; Allen & Myers, 2019). The current study had a Cronbach’s alpha of 0.75 for the twenty questions scored.

### Procedure

Participants signed up for individual testing sessions using an online system (SONA systems). They provided written informed consent at the start of their testing session and completed a demographics questionnaire that included gender, age, race/ethnicity, and education level. The order of the personality inventories and the avatar task were counter-balanced between participants. All participants completed the two scenarios in the avatar task in the same order. Following the completion of the avatar task, the participants completed a post-questionnaire that asked about the how they felt about their satisfaction in the choices in the selection of their specific avatar task, how much the avatar represented them, and how much prior computer game experience they had. All procedures were approved by the Institutional Review Board of the University of Northern Colorado (approval 799609-4) and conformed to guidelines established by the Declaration of Helsinki and the U.S. Federal Government.

### Data analysis

First, sample characteristics and gender differences in Type D, NA, SI and avatar avoidance scores were examined with independent measures t-tests. Effect sizes were calculated as Hedges’ g.

Second, Type D was analyzed as a continuous variable. Pearson’s product moment correlations were calculated to analyze the relationships between the Type D scores including the SI and NA subscales and avoidance scores on the avatar task. Bonferroni adjustments for repeated Pearson’s r resulted in a significance threshold of 0.0167. Fisher r-to-z transformations were calculated to test for significant differences between correlation coefficients. Relationships were also analyzed by stepwise linear regressions were with predictors of gender, age, education level, and avatar avoidance scores. Dependent variables were Type D, SI, and NA scores.

Third, Type D was also analyzed as a dichotomous variable in that individuals were categorized into four groups consisting of those with low scores for both SI and NA, those with high scores for only SI, those with high scores for only NA, and those with high scores for both SI and NA (*i.e*., Type D). A two-way ANOVA with post-hoc Tukey HSD comparisons was calculated to test for differences in the avatar avoidance scores (DV) between the IVs of gender (males and females) and Type D group membership (Type D, high SI, high NA, and low both SI and NA). Effect sizes for ANOVAs were reported as eta squared (η^2^).

## Results

### Descriptive statistics and gender differences

The mean Type D, S, NA scores and avatar avoidance scores as well as gender comparisons are shown in Table 1. There were no significant gender effects for Type D (*p* > 0.31), SI (*p* > 0.38), and NA (*p* = 0.10). Females did exhibit higher avatar avoidance scores than males (*t* (300) = 2.01, *p* = 0.02, *g* = 0.257).

**Table 1:** Gender comparisons for Type D, SI, NA, and avatar avoidance scores.

	Total mean (*SD*)	Female mean (*SD*)	Male mean (*SD*)	Gender effect significance level
Type D	134.5 (124.6)	139.6 (124.7)	124.3 (135.9)	*p* = 0.31
SI	11.7 (5.7)	11.9 (5.7)	11.3 (5.5)	*p* = 0.38
NA	10.1 (5.7)	10.5 (5.7)	9.4 (5.9)	*p* = 0.10
Avatar avoidance	18.6 (5.7)	19.0 (5.7)	17.6 (5.1)	*p* = 0.02

**Note:**

SD, standard deviation.

### Type D analyzed as a continuous variable

**Figure 2:**
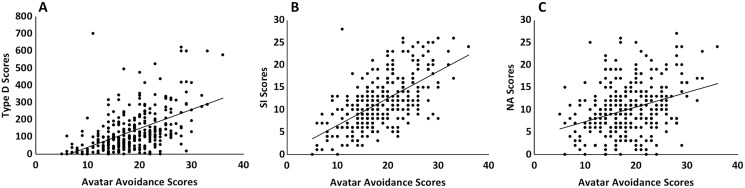
The relationships between avatar avoidance scores and Type D, SI, and NA scores. Avatar avoidance scores had a moderate positive relationship to (A) Type D scores (*r* = 0.50), (B) SI scores (*r* = 0.61), and (C) NA scores (*r* = 0.33). Type D and SI scores had significantly stronger relationships to avatar avoidance scores than did NA.

The relationships between the Type D, SI, NA, and avatar avoidance scores are shown in Table 2. Type D scores had a strong positive relationship with both SI and NA scores. Avatar avoidance scores had moderate positive relationships to Type D, SI, and NA scores as plotted in Fig. 2. Fisher r-to-z transformations revealed that the relationships between avatar avoidance scores and Type D and SI did not significantly differ (*p* = 0.06). However, the correlation between avatar avoidance scores and NA scores was significantly lower than those for Type D (*z* = −4.48, *p* < 0.001) and SI (*z* = −2.57, *p* = 0.01).

**Table 2:** Inter-correlations between Type D, SI, NA, and avatar avoidance scores.

	Type D	SI	NA	Avatar avoidance
Type D	–			
SI	0.81*	–		
NA	0.85*	0.46*	–	
Avatar avoidance	0.50*	0.61*	0.33*	–

**Notes:**

* *p* < 0.001.

The predictors of Type D, SI, and NA scores were further analyzed with a backward stepwise linear regression with gender, age, education level and avatar avoidance scores as predictors. As shown in Table 3, this analysis revealed that Type D could be best predicted by a model that included avatar avoidance scores and education level (R = 0.52, R^2^ = 0.27, F (2, 299) 55.51, *p* < 0.001). SI could also be best predicted by a model that included avatar avoidance scores and education level (R = 0.62, R^2^ = 0.38, F (2, 299) = 94.6, *p* < 0.001). NA could be best predicted by a model that included avatar avoidance scores (R = 0.33, R^2^ = 0.11, F (1, 300) = 36.09, *p* = 0.004).

**Table 3:** Details on regression analyses predicting Type D, SI, and NA scores.

Best predictors of Type D scores					
Variables	Standardized β	b	Standard error	*p* value	*R* ^*2*^
Education level	0.144	14.7	5.05	0.004	0.27
Avatar avoidance	0.510	11.1	1.08	<0.001	
Constant		−261.8	69.71	<0.001	
Best predictors of SI scores					
Variables	Standardized β	b	Standard error	*p* value	*R* ^*2*^
Education level	0.134	0.620	0.210	0.003	0.38
Avatar avoidance	0.614	0.610	0.044	<0.001	
Constant		−7.69	2.90	<0.001	
Best predictors of NA scores					
Variables	Standardized β	b	Standard error	*p* value	*R* ^*2*^
Avatar avoidance	0.327	0.328	0.055	<0.001	0.11
Constant	0	4.03	1.06	<0.001	

### Type D analyzed as a dichotomous variable

Based on a cut-off score of ten for both SI and NA, one-hundred seventeen participants (38.7%) were categorized as Type D. Sixty-eight participants (22.5%) were categorized as high SI. Thirty-seven participants (12.3%) were categorized as high NA. There were eighty participants (26.5%) categorized as scoring low on both SI and NA.

**Figure 3:**
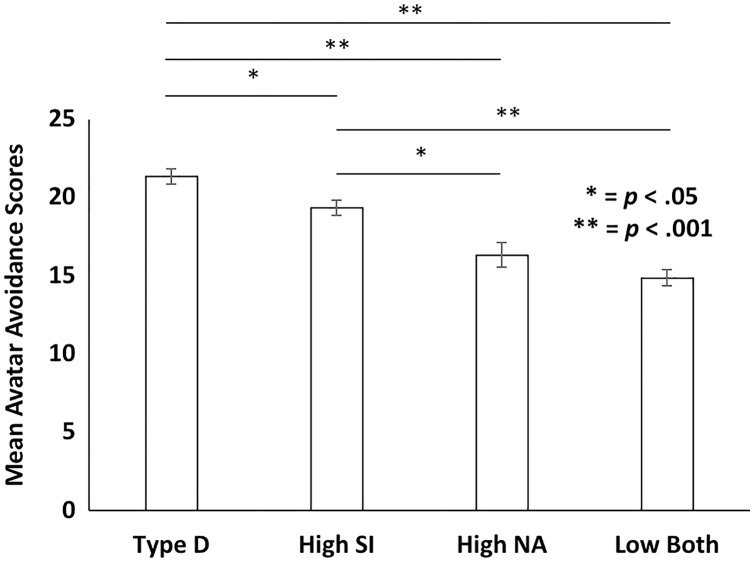
Mean avatar avoidance scores for the Type D, high SI, high NA, and low both SI and NA groups. Overall, there was a significant difference in avatar avoidance scores across the four groups. Specifically, the Type D group had higher avoidance scores than the high SI, high NA, and low both SI and NA groups. The high SI group had higher avoidance scores than the high NA and low both SI and NA groups.

The effects of gender (female and male) and level of Type D (Low Both, High SI, High NA, Type D) on avatar avoidance scores were analyzed with a two-way ANOVA. As shown in Fig. 3, analysis revealed a main effect of group *F* (3, 294) = 29.32, *p* < 0.001, *η*^2^ = 0.23. *Post hoc* analyses with Tukey HSD pairwise comparisons revealed that the Type D group had significantly higher avoidance scores on the avatar task than did the high SI group (*p* = 0.049), the high NA (*p* < 0.001), and the low NA and SI group (*p* < 0.001). The high SI group also had significantly higher avoidance scores on the avatar task than did the high NA group (*p* = 0.018) and the group with low SI and NA scores (*p* < 0.001). The high NA group did not differ from the low SI and NA group (*p* = 0.46).

There was also a main effect of gender *F* (1, 294) = 5.14, *p* = 0.024, *η*^2^ = 0.017 such that females had higher avatar avoidance scores than males. There was no interaction between gender and levels of DS-14 scores *F* (1, 294) = −0.023, *p* = 1, *η*^2^ = −0.0024.

## Discussion

The aim of the current study was to explore the role of avoidance in Type D with a computer-based avatar task designed to assess behavioral inhibition while interacting with strangers in social situations. The results of the current study fit within the parameters of previously published studies. The mean Type D score is slightly higher than the percentages reported for cardiac patients classified as Type D in previous research (Denollet, Sys & Brutsaert, 1995; Denollet et al., 1996; Denollet, Vaes & Brutsaert, 2000; Whitehead et al., 2007) and somewhat consistent for undergraduate samples (Allen, 2018; Polman, Borkoles & Nicholls, 2010; Gupta & Basak, 2013). There were no significant gender effects for Type D, SI or NA scores in the current study which is consistent with some prior reports (*e.g*., Denollet, 2005; Williams et al., 2008; Barnett et al., 2009; Vukovic et al., 2014). Overall, the mean scores for the avatar task fit within the range of scores on the avatar task previously reported for undergraduate samples (*e.g*., Myers et al., 2016a; Allen, 2018). There was a small, but significant, gender effect in that females had higher avatar avoidance scores than males which had not been reported in prior studies (Myers et al., 2016a, 2016b; Allen, Jameson & Myers, 2017; Allen, 2018; Allen & Myers, 2019). This novel finding of a gender effect for the avatar task may be due in part to the current study having the largest sample yet tested with the avatar task as well as having a mainly female sample. Future work should attempt to achieve large samples sizes with a more balanced female to male ratio to further examine the role of gender in avoidance on the avatar task.

Type D was first examined as a continuous variable. We hypothesized that avoidance scores on the avatar task would be positively related to Type D and SI scores. The finding that avoidance scores and Type D and SI scores had a moderate positive relationship supported this hypothesis. The avatar task was developed to assess BI which we have discussed is similar to or a component of SI. The strength of the relationship between avoidance scores on the avatar task and SI fell within the range of correlations previously reported for BI and performance on the avatar task (Allen, Jameson & Myers, 2017; Myers et al., 2016a). This finding supports the relationship between SI and BI. We also hypothesized that avatar avoidance scores would not be related to NA scores. This hypothesis was not supported by the current findings of a moderate positive relationship between NA and avatar avoidance score. However, the relationship between the avatar avoidance and NA scores was significantly weaker than the relationships between avoidance and the Type D and SI scores. Thus, these findings support the idea that avoidance is more related to Type D personality and SI than NA.

Avoidance scores as well as variables such as gender, age, and educational level were also tested as possible predictors of Type D, SI, and NA scores. Regression analyses revealed that Type D and SI scores were best predicted by avoidance scores on the avatar task and level of education, but not gender or age. In addition, NA scores were best predicted by avoidance scores on the avatar task, but not gender, age, or education level. Avoidance scores on the avatar task were the only consistent predictor for these three scores which fits with the correlational results. Type D and SI scores, but not NA scores, were also predicted by educational level which was surprising in that there was no corresponding effect of age. However, there is limited evidence for an education effect for Type D in that Type D status has been found to be related to lower education levels (van Bon-Martens et al., 2012) but also higher education levels (Klaassen et al., 2012; Kelpis et al., 2013). However, these studies compared higher education or mid-level vocational education to those with lower education levels. The range of education levels in current study is more limited given that all students were within the range of undergraduates from first semester college freshmen to students who have completed 6 years of college. Future studies should continue to explore how demographic characteristics of participants may play a role in Type D personality.

The findings of a moderate positive relationship between avoidance and Type D are supported by a literature which has examined avoidant coping styles and Type D personality (Polman, Borkoles & Nicholls, 2010; Martin et al., 2011; Williams & Wingate, 2012). Specifically, Polman, Borkoles & Nicholls (2010) reported that Type D individuals exhibit more passive and maladaptive avoidance coping strategies such as resignation and withdrawal as measured by the Brief Approach/Avoidance Coping Questionnaire (BACQ; Finset et al., 2002). Type D individuals were also less likely to utilize more adaptive approach coping mechanisms and had higher levels of perceived stress (Cohen, Kamarck & Mermelstein, 1983). These findings are in strong agreement with those of Allen (2021) which found that passive avoidance, specifically resignation and withdrawal coping, had a moderate positive relationship to avatar avoidance scores. In addition, this study reported that avatar avoidance scores had a moderate negative relationship to approach coping styles as well as a moderate positive relationship to perceived stress. Thus, Type D and avoidance on the avatar task share similar relationships to avoidance and approach coping styles as well as perceived stress.

Furthermore, Martin et al. (2011) found that Type D individuals relied more often on avoidant forms of coping and less often on positive/problem focused strategies or social support to cope. Avoidant coping styles in this study included behavioral disengagement, denial, self-blame, and self-distraction, substance use, and venting as measured by the Brief COPE inventory (Carver, 1997). Similarly, Williams & Wingate (2012) reported that Type D was positively correlated with avoidant coping as measured by the COPE Inventory (Carver, Scheier & Weintraub, 1989) consisting of behavioral disengagement, denial, mental disengagement, and substance. Dunkley et al. (2012) also reported that both SI and NA were positively correlated with avoidant coping was measured by the behavioral disengagement, denial, and mental disengagement scales of the COPE inventory. The findings of these studies fit with those of Allen & Myers (2019) which examined the relationships of performance on the avatar task with avoidant coping styles including behavioral disengagement, denial, self-blame, self-distraction, and substance use grouped together as an aggregate avoidance factor. Overall, avoidance scores on the avatar task had a positive relationship to this avoidance coping aggregate score. More specifically, avoidance scores on the avatar task had a positive relationship to the avoidant coping styles of behavioral disengagement, denial, self-blame and a negative relationship to the non-avoidant coping styles of active coping, humor, and positive reframing. Thus, there is strong agreement between the relationships of Type D personality and avoidance on the avatar task and avoidant coping styles.

Relationships between avoidance and SI are also supported by recent studies. Duijndam et al. (2021) examined the relationships between avoidance and SI and its three facets of behavioral inhibition, interpersonal sensitivity, and social withdrawal. They found that SI behavioral inhibition and social withdrawal, but not interpersonal sensitivity, were associated with an avoidant style of situational emotional regulation. However, Duijndam et al. (2020) found that social inhibition and two of its components behavioral inhibition and social withdrawal were not affected by avoidance tendencies in a facial version of the Approach-Avoidance Task (AAT; Wiers et al., 2009). Based on the lack of effect for avoidant tendencies with facial stimuli, Duijndam et al. (2020) hypothesized that avoidant tendencies of SI individuals may be limited to real interpersonal settings. It would be of interest to test this hypothesis with avoidance measured in a virtual setting such as the computer-based avatar avoidance task. Future studies examining the relationships between avoidance and SI should also include the three facets of behavioral inhibition, interpersonal sensitivity, and social withdrawal measured by the Social Inhibition Questionnaire (SIQ-15; Denollet & Duijndam, 2019) which could further the understanding of how these various aspects of SI are related to avoidance as measured by the avatar avoidance task. There is also evidence for a relationship between avoidance and NA. For example, Eaton & Bradley (2008) found that NA predicted the use of avoidance and emotion-focused coping strategies while Hildebrandt & Hayes (2012) reported that NA had a strong positive relationship with experiential avoidance, specifically a tendency to avoid difficult thoughts and feelings. Overall, there is support in the literature for the current findings that avoidance, as measured by the avatar task, is positively related to and predictive of Type D personality as well as SI, and NA scores.

Avoidance was also examined with Type D operationalized as a dichotomous variable in which participants were categorized into Type D, high SI, high NA and low SI and NA groups based on standard cut-off scores. Overall, there was a large effect of Type D group membership such that avatar avoidance scores significantly differed between these four groups. Specifically, Type D individuals had higher avatar avoidance scores than did the three other groups. Furthermore, high SI individuals had higher avatar scores than did the high NA and both low SI and NA groups. These findings fit with prior work that found the standard cut-off scores established for BI and harm avoidance (HA) result in significant differences in avatar avoidance scores (Allen, Jameson & Myers, 2017). Overall, the findings that the relationship between avoidance and Type D is consistent whether Type D is analyzed as a continuous or a dichotomous variable supports the applicability of the avatar task to the study of Type D and its related subscales. The current findings also add distressed (Type D) personality to a growing list of avoidant-related temperaments including BI, HA, behavioral avoidance, and avoidant coping styles that have positive relationships to avoidance scores on the avatar task.

In addition to its relevance in assessing several forms of avoidant tendencies, the avatar task may also provide a novel benefit for the assessment of avoidance in those expressing Type D personality beyond the current self-report inventories. The avatar task was developed as an alternative to paper-and-pencil self-report inventories such as the AMBI (Gladstone & Parker, 2005) for measuring BI. An additional alternative to paper and pencil self-report was put forth by Vazire & Mehl (2008) who proposed the use of peer ratings as a way to reduce potential biases of self-ratings. The use of the ratings of others was echoed by Myers et al. (2016a) who suggested behavioral reports from a family member or friend could serve as a comparison for paper and pencil inventories and the avatar task. However, real world observations may be difficult, especially in the case of avoidant individuals including those expressing Type D who may be resistant to revealing their tendencies towards SI and NA to family, friends, and medical professionals. Myers et al. (2016a) suggested that an approximation to this ideal may be interactive virtual environments such as the current avatar task, which allow the user to experience simulated situations *via* an “avatar,” a graphical representation of the user whose behavior the user controls (Blascovich et al., 2002). The interactive virtual environment of the avatar task may also be a more ecologically valid measure of avoidance than a paper and pencil questionnaire. The use of the computer-based avatar task as an addition to the DS-14 paper and pencil inventory could be of special benefit if adapted for use in telemedicine where the individual could complete the avatar task at home *via* internet connection. Telemedicine has increased significantly in popularity and use during the current COVID-19 pandemic as an alternative for in person assessment. Myers et al. (2016a) also suggested that virtual environments might be developed as therapeutic tools for those with exaggerated avoidance, as a component of exposure therapy to help participants explore how they might change their behavior within the context of a virtual environment. The initial findings in the current study or the relevance of the avatar avoidance task to Type D personality supports further work addressing how the avatar task is related to more traditional self-report assessments of Type D and SI.

### Limitations and future work

One limitation of the current study is that it tested a non-clinical population of mainly young, healthy, college undergraduates. Type D personality has been explored mainly in older clinical populations, specifically cardiac and cancer patients, and individuals reporting symptoms of PTSD, anxiety disorders, and depression. While the avatar task has been successfully applied to a population with self-reported PTSD symptoms (Myers et al., 2016b), it has yet to be investigated with other clinical populations. It would be of interest to extend the current work with the avatar task to patient populations who express distressed (Type D) personality. The current study also has limited generalizability of findings due to the utilization of samples from one undergraduate institution which had a majority of female participants.

Future work should replicate with an independently collected sample that includes a range of ages that better approximate the middle aged and elderly patients in typical Type D studies (*e.g*., Denollet, 2005; Kupper et al., 2013a). Future longitudinal studies utilizing both paper-and-pencil inventories and the avatar task can also investigate if BI and tendencies for exaggerated avoidance earlier in life could predict later development of Type D personality, or avoidance-related psychopathologies such as PTSD and anxiety disorders.

## Conclusions

The current study tested a computer-based avatar task designed to measure avoidant tendencies in social situations with individuals assessed for Type D personality. Type D personality was examined as both a continuous and dichotomous variable. Performance on the avatar avoidance task had a moderate positive relationship with Type D and SI scores and to a lesser degree NA scores. Additionally, Type D individuals had higher avoidance scores on the avatar task than non-Type D individuals. Taken together, these results indicate that avatar task has value as an objective measure of avoidance behaviors in Type D individuals which could assist in the identification of cardiac patients who may be at risk for poor health outcomes following major medical procedures due to the interactions of social inhibition, negative affectivity, and maladaptive avoidant coping styles or strategies. A novel benefit of the computer-based avatar avoidance task is that it may have more ecological validity than a paper and pencil questionnaire in that it assesses how an individual would respond/avoid in a virtual social setting. Future work should test the utility of the avatar task in clinical populations to determine if the current findings from a non-clinical undergraduate sample generalize to individual diagnosed with distressed (Type D) personality.

## Supplemental Information

10.7717/peerj.14302/supp-1 Supplemental Information 1 Demographics and scores for Type D, NA, SI, BI and the avatar task.

## Additional Information and Declarations

### Data Availability

The following information was supplied regarding data availability: The raw data is available as a Supplemental File.
